# Risk Factors for Being Seronegative following SARS-CoV-2 Infection in a Large Cohort of Health Care Workers in Denmark

**DOI:** 10.1128/Spectrum.00904-21

**Published:** 2021-10-20

**Authors:** Caroline Klint Johannesen, Omid Rezahosseini, Mikkel Gybel-Brask, Jonas Henrik Kristensen, Rasmus Bo Hasselbalch, Mia Marie Pries-Heje, Pernille Brok Nielsen, Andreas Dehlbæk Knudsen, Kamille Fogh, Jakob Boesgaard Norsk, Ove Andersen, Claus Antonio Juul Jensen, Christian Torp-Pedersen, Jørgen Rungby, Sisse Bolm Ditlev, Ida Hageman, Rasmus Møgelvang, Ram B. Dessau, Erik Sørensen, Lene Holm Harritshøj, Fredrik Folke, Curt Sten, Maria Elizabeth Engel Møller, Frederik Neess Engsig, Henrik Ullum, Charlotte Sværke Jørgensen, Sisse R. Ostrowski, Henning Bundgaard, Kasper Karmark Iversen, Thea Kølsen Fischer, Susanne Dam Nielsen

**Affiliations:** a Department of Clinical Research, Nordsjaellands Hospital, Hilleroed, Denmark; b Viro-immunology Research Unit, Department of Infectious Diseases, Rigshospitalet, University of Copenhagen, Copenhagen, Denmark; c Department of Clinical Immunology, Rigshospitalet, Copenhagen, Denmark; d Department of Cardiology, Herlev og Gentofte Hospital, University of Copenhagen, Herlev, Denmark; e Department of Emergency Medicine, Herlev og Gentofte Hospital, University of Copenhagen, Herlev, Denmark; f Department of Clinical Medicine, University of Copenhagen, Copenhagen, Denmark; g Department of Cardiology, Rigshospitalet, University of Copenhagen, Copenhagen, Denmark; h Department of Emergency, Copenhagen University Hospital – Amager og Hvidovre, Hvidovre, Denmark; i Department of Clinical Research, Copenhagen University Hospital – Amager og Hvidovre, Hvidovre, Denmark; j Department of Clinical Biochemistry, Copenhagen University Hospital – Nordsjællands Hospital, Hillerød, Denmark; k Department of Cardiology and Clinical Research, Nordsjællands Hospital, Hillerød, Denmark; l Department of Cardiology, Aalborg University Hospital, Aalborg, Denmark; m Department of Public Health, University of Copenhagen, Copenhagen, Denmark; n Department of Endocrinology, Copenhagen University Hospital – Bispebjerg and Frederiksberg, Bispebjerg, Copenhagen, Denmark; o Copenhagen Center for Translational Research, Copenhagen University Hospital – Bispebjerg and Frederiksberg, Bispebjerg, Copenhagen, Denmark; p Department of Pulmonary Medicine, Copenhagen University Hospital – Bispebjerg and Frederiksberg, Bispebjerg, Copenhagen, Denmark; q Mental Health Services-The Capital Region of Denmark, Copenhagen, Denmark; r Department of Clinical Microbiology, Zealand University Hospital–Slagelse, Slagelse, Denmark; s Department of Regional Health Research, University of Southern Denmark, Odense, Denmark; t Copenhagen University Hospital –Copenhagen Emergency Medical Services, Copenhagen, Denmark; u Diagnostisk Enhed, Copenhagen University Hospital, Bornholm, Denmark; v Department of Infectious Disease, Copenhagen University Hospital –Amager and Hvidovre, Hvidovre, Denmark; w Statens Serum Institut, Copenhagen, Denmark; x Department of Public Health, Global Health Section, University of Copenhagen, Denmark; Karolinska Institutet

**Keywords:** asymptomatic infections, body mass index, health care workers, risk factor, SARS-CoV-2, seroconversion

## Abstract

Most individuals seroconvert after infection with severe acute respiratory syndrome coronavirus 2 (SARS-CoV-2), but being seronegative is observed in 1 to 9%. We aimed to investigate the risk factors associated with being seronegative following PCR-confirmed SARS-CoV-2 infection. In a prospective cohort study, we screened health care workers (HCW) in the Capital Region of Denmark for SARS-CoV-2 antibodies. We performed three rounds of screening from April to October 2020 using an enzyme-linked immunosorbent assay (ELISA) method targeting SARS-CoV-2 total antibodies. Data on all participants’ PCR for SARS-CoV-2 RNA were captured from national registries. The Kaplan-Meier method and Cox proportional hazards models were applied to investigate the probability of being seronegative and the related risk factors, respectively. Of 36,583 HCW, 866 (2.4%) had a positive PCR before or during the study period. The median (interquartile range [IQR]) age of 866 HCW was 42 (31 to 53) years, and 666 (77%) were female. After a median of 132 (range, 35 to 180) days, 21 (2.4%) of 866 were seronegative. In a multivariable model, independent risk factors for being seronegative were self-reported asymptomatic or mild infection hazard ratio (HR) of 6.6 (95% confidence interval [CI], 2.6 to 17; *P* < 0.001) and body mass index (BMI) of ≥30, HR 3.1 (95% CI, 1.1 to 8.8; *P* = 0.039). Only a few (2.4%) HCW were not seropositive. Asymptomatic or mild infection as well as a BMI above 30 were associated with being seronegative. Since the presence of antibodies against SARS-CoV-2 reduces the risk of reinfection, efforts to protect HCW with risk factors for being seronegative may be needed in future COVID-19 surges.

**IMPORTANCE** Most individuals seroconvert after infection with severe acute respiratory syndrome coronavirus 2 (SARS-CoV-2), but negative serology is observed in 1 to 9%. We found that asymptomatic or mild infection as well as a BMI above 30 were associated with being seronegative. Since the presence of antibodies against SARS-CoV-2 reduces the risk of reinfection, efforts to protect HCW with risk factors for being seronegative may be needed in future COVID-19 surges.

## INTRODUCTION

Severe acute respiratory syndrome coronavirus 2 (SARS-CoV-2) infection has as of June 2021 affected more than 176 million individuals and caused more than 3.8 million deaths worldwide (1). Both the humoral and the cellular immune systems react to SARS-CoV-2 infection, and the vast majority of individuals infected by SARS-CoV-2 have detectable antibodies 3 weeks after infection (2, 3), but 1 to 9% are seronegative (3, 4). It has been shown that the presence of anti-spike or anti-nucleocapsid IgG antibodies reduces the risk of SARS-CoV-2 reinfection (5, 6). As such, it is important to determine the risk factors for being seronegative. We aimed to investigate the risk factors for being seronegative following PCR-confirmed SARS-CoV-2 infection in a large prospective cohort of health care workers (HCW) in Denmark (7).

## RESULTS

Of 36,583 HCW who participated in any of the three rounds of screening, 866 (2.4%) had a positive PCR test and were included in this study. Participants contributed with 94,377 person-days of follow up with a median (interquartile range [IQR]) of 132 (35 to 180) days of follow-up per HCW. The median (IQR) age was 42 (31 to 53) years, and 666 (77%) were female (Table 1).

**TABLE 1:** Characteristics of the participants in total and divided by serostatus

Characteristic	*n* (%) (*N* = 866)	ELISA seropositive [*n* (%)] (*N* = 845)	ELISA seronegative [*n* (%)] (*N* = 21)
Age			
<40	380 (44)	369 (44)	11 (52)
40–60	385 (44)	376 (45)	9 (43)
>60	101 (12)	100 (12)	1 (4.8)
Sex			
Female	666 (77)	647 (77)	19 (90.5)
Male	200 (23)	198 (23)	2 (9.5)
BMI			
<25	423 (49)	415 (49)	8 (38)
25–30	241 (28)	234 (28)	7 (33)
30	109 (13)	103 (12)	6 (29)
No information	93 (11)	93 (11)	0 (0)
Smoking			
Yes	96 (11)	95 (11)	1 (4.8)
No	754 (87)	734 (87)	20 (95.2)
No information	16 (1.9)	16 (1.9)	0 (0)
Reported having contact with patients			
Yes	810 (93.5)	790 (93.5)	20 (95.2)
No	55 (6.4)	54 (6.4)	1 (4.8)
No information	1 (0.1)	1 (0.1)	0 (0)
Severity of the disease			
No clinical symptom or clinically symptomatic but quite well at home	253 (29)	238 (28)	15 (71)
Clinically symptomatic and bedridden at home or at hospital	540 (62)	534 (63)	6 (29)
No information	73 (8.4)	73 (8.6)	0 (0)
Median time since PCR (days)	131	136	80

The median (IQR) time between a positive PCR and an enzyme-linked immunosorbent assay (ELISA) antibody test was 18 (14 to 30), 55 (42 to 64), and 172 (153 to 187) days for screening rounds 1, 2, and 3, respectively. A total of 483 (56%) out of 866 HCW participated in more than one screening round, and 116 (13%) participated in all three rounds. Of the 866 HCW, 21 (2.4%) had no detectable antibodies at last follow-up.

Among participants who all had a positive PCR, 540/866 (62%) reported having had symptomatic SARS-CoV-2 infection, and 253/866 (29%) reported no or mild symptoms.

One participant was seronegative at the first test after a positive PCR (66 days after PCR) but seropositive at the second test (182 days after PCR). We only included the first test after PCR if it was more than 9 days after the positive PCR test. Three participants did not meet the time-between-test cutoff but were seronegative shortly after their PCR-test and seropositive in the following serum samples tested.

### Risk factors for being seronegative.

In a univariable Cox proportional hazards model, participants who had self-reported mild or no symptoms had a significantly increased risk of being seronegative with a hazard ratio (HR) of 7.0 (95% confidence interval [CI], 2.7 to 18; *P* < 0.001) compared to participants who were clinically symptomatic. Participants with a body mass index (BMI) above 30 kg/m^2^ versus participants with a BMI lower than 25 kg/m^2^ had an HR of 3.0 (95% CI, 1.0 to 8.6; *P* = 0.043) for being seronegative. In a multivariate Cox proportional hazards model, including both BMI and severity of disease, we found almost the same results (Table 2).

**TABLE 2:** Risk factors for negative anti-SARS-CoV-2 antibody in adjusted and unadjusted Cox regression models^*a*^

Characteristic	Unadjusted HR (95% CI)	*P* value	Adjusted HR (95% CI)	*P* value
BMI				
<25	1.0 (reference)			
25–30	1.4 (0.50 to 3.8)	0.618	1.4 (0.52–4.0)^*b*^	0.484
>30	3.0 (1.0–8.6)	0.043	3.1 (1.1–8.8)^*c*^	0.039
Severity of disease				
Clinically symptomatic and bedridden at home or at hospital	1.0 (reference)			
No clinical symptom or clinically symptomatic but quite well at home	7.0 (2.7–18)	<0.001	6.6 (2.6–17)^*c*^	<0.001

a BMI, body mass index; CI, confidence interval; HR, hazard ratio.

b Adjusted for severity of disease and age.

c Adjusted for BMI category and age.

**FIG 1:**
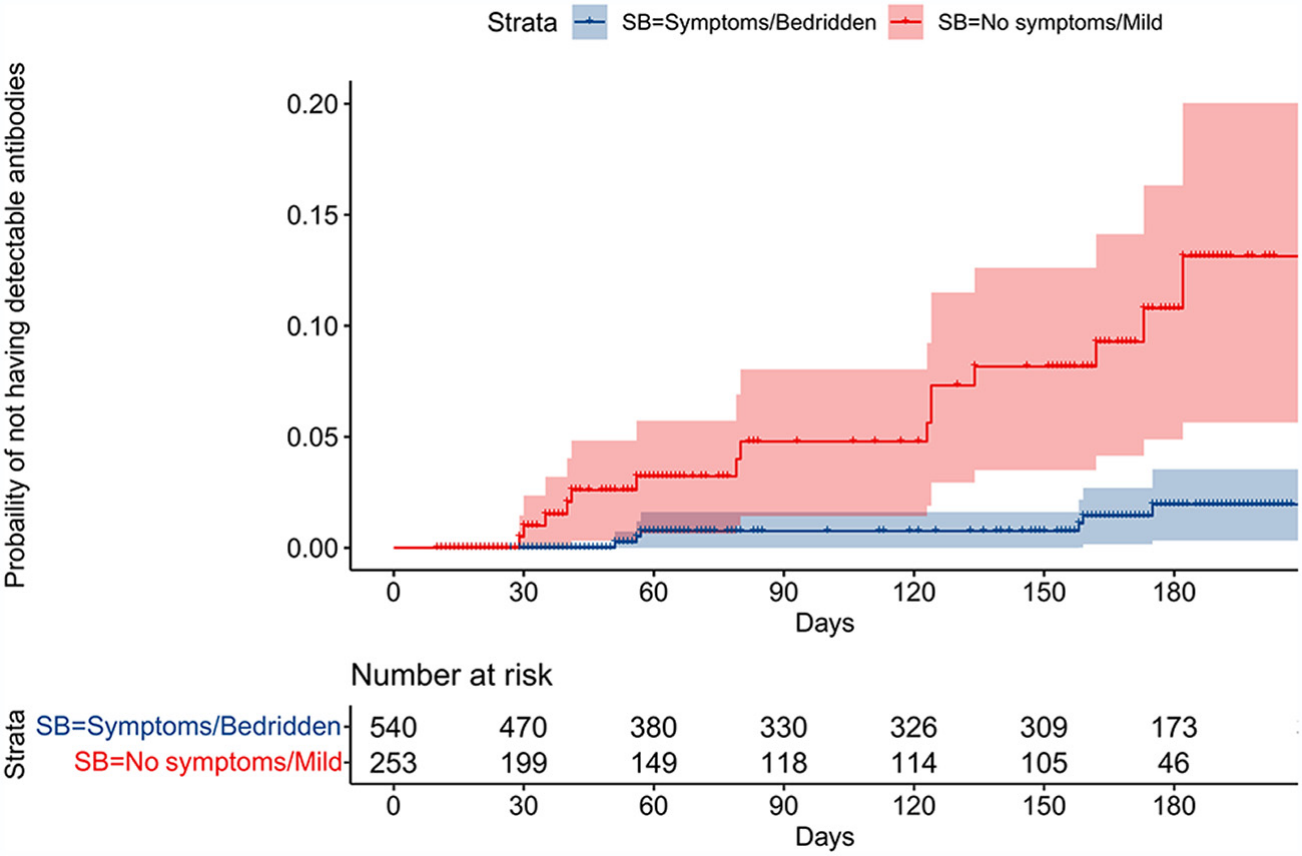
Kaplan-Meier plot for probability of being seronegative over time. The risk of being seronegative is higher among participants with no symptoms or mild symptoms, and the difference in risk increases over time. After 200 days of follow up, the risk is estimated to be 13% (95% CI, 5.7 to 20) in participants with no or mild symptoms and 1.9% (95% CI, 0.7 to 3.5) in participants bedridden at home or at hospital.

Figure 1 presents the probability of being seronegative over time since positive PCR test, stratified by self-reported level of symptoms in two categories, no clinical symptom or mild clinical symptoms and clinically symptomatic and bedridden at home or at hospital. The risk of being seronegative is higher among participants with no symptoms or mild symptoms, and the difference in risk increases over time. After 200 days of follow-up, the risk is estimated to be 13% (95% CI, 5.7 to 20) in participants with no or mild symptoms and 1.9% (95% CI, 0.7 to 3.5) in participants bedridden at home or at hospital.

## DISCUSSION

In this large cohort of HCW with PCR-confirmed SARS-CoV-2 infection, we found that only 2.4% were seronegative. Asymptomatic or mildly symptomatic SARS-CoV-2 infection and obesity were associated with being seronegative.

Compared to symptomatic HCW, those who were asymptomatic or had mild symptoms were at almost seven times higher risk of being seronegative, corroborating previous reports (8–11). It has been reported that severity of COVID-19 affects the initial magnitude but not persistence of antibodies (3), Although a recent study demonstrated that antibodies decline over time and their half-life is positively associated with the intensity of the initial response (12). We do not present data implying a correlation between initial antibody response and antibody persistence, as we did not measure antibody titers initially or at the following time points. These controversies could be partly due to different ELISAs that were applied. Unlike traditional indirect ELISAs (antigen-antibody-antibody), antigen sandwich assay (antigen-antibody-antigen) uses a labeled virus antigen instead of labeled secondary anti-human antibody for detection, so that each anti-SARS-CoV-2 antibody bridges two recombinant antigens of the virus that affect the sensitivity of the test (13).

It has been shown that the presence of antibodies against other seasonal human coronaviruses (other than SARS-CoV-2) can protect against a severe course of COVID-19 (14). We did not have information about prior infection with other human coronaviruses, but because the severity of COVID-19 is associated with risk of being seronegative, it seems relevant to adjust results in future studies. In addition, smoking is associated with COVID-19 severity and progression (15), although we could not find a significant association between smoking status and being seronegative.

In comparison with a BMI of <25 kg/m^2^, participants with a BMI of >30 had a three times higher risk of being seronegative even after adjustment for severity of the disease. Obesity causes leptin and insulin resistance and is accompanied by systemic low-grade inflammation, causing high levels of proinflammatory cytokines and a hampered cellular response to viral infections (16). A diminished response can thus also be the case with patients with obesity and SARS-CoV-2-infection, thus leading to increased awareness of the risk of reinfection. Policy makers could consider this for vaccination or revaccination of HCWs. Furthermore, it is worth mentioning that there could be residual confounding factors that we did not adjust for.

The large sample size, extensive follow-up, and prospective gathering of data were strengths of our study. Furthermore, we had access to complete data on SARS-CoV-2 PCR from the Danish Microbiology Database (MiBa), which contains all data on microbiological samples in Denmark. Finally, we used the Wantai ELISA, which is one of the most sensitive assays available (17). However, Wantai ELISA is an antigen sandwich assay, and the results may only be applicable to antigen bridging assays and only for a limited time interval. Other assays could produce different results. In addition, the study was limited to HCW, and therefore, we did not have any elderly in the population or participants who were chronically too ill to work. This meant that there were almost no cases of SARS-CoV-2 hospitalizations in the data set and no cases of either admission to intensive care units or death in our study population. Furthermore, the information on participants was collected at the time of inclusion in the study population. The survey did not include questions about immunodeficiency conditions or any metabolic or systemic disturbance factors.

In conclusion, 2.4% of HCW were seronegative after PCR confirmed COVID-19 infection. Asymptomatic or mild infection and a BMI higher than 30 were associated with being seronegative. It has been shown that presence of antibodies considerably reduces the risk of SARS-CoV-2 reinfection, and efforts to protect HCW with these risk factors in subsequent COVID-19 surges may be needed.

## MATERIALS AND METHODS

### Study design and participants.

We used data from a prospective cohort study, where all HCW and other staff employed at hospitals and primary care facilities in the Capital Region of Denmark were invited to participate in a screening program for SARS-CoV-2 antibodies. As previously described (7), screening was offered in three rounds (from early April to early October 2020). All participants provided blood samples for SARS-CoV-2 serology and were asked to fill in a questionnaire on exposures, risk factors, and symptoms of COVID-19. Participants who provided blood at least once after a positive PCR were included into the analyses. The study was registered with the Danish Data Protection Authorities (P-2020-361) and presented to the regional scientific ethics committee of the Capital Region (Jnr-H-20026288), which concluded that the study did not require a scientific ethical approval. The study was registered at ClinicalTrials.gov under registration no. NCT04346186.

### SARS-CoV-2 antibody measurement using ELISA.

SARS-CoV-2 total antibodies were measured qualitatively by a double-antigen bridging enzyme-linked-immunosorbent assay (ELISA) targeting the SARS-CoV-2 receptor-binding domain (S-RBD) (Wantai BioPharm, Beijing, China) according to the manufacturer’s instructions. A signal/cutoff ratio of ≥1/1 was interpreted as positive. This ELISA was previously validated yielding a sensitivity of 96.7% and a specificity of 99.5% (17).

### SARS-CoV-2 PCR-testing.

During the study period, HCW were advised to test at regular intervals with or without symptoms. Results from the general Danish PCR-testing program were captured from the Danish Health Data Agency and linked to participants through the Civil Registration System, using the unique personal identification number as described elsewhere (18). Real-time PCR (RT-PCR) analyses for SARS-CoV-2 RNA were performed at Statens Serum Institute (SSI) and TestCenter Denmark using primers targeting the E gene. A test was defined as positive if the cycle threshold value was ≤38. The results from these tests were made available to researchers in a categorical format (positive, negative, never tested)

### Definitions.

To ensure sufficient time from infection to seroconversion, we included ELISA results only if blood sampling was done more than 9 days after a positive PCR test (2).

Disease severity was self-reported as no or mild symptoms (no clinical symptom or clinically symptomatic but quite well) and symptomatic (clinically symptomatic and bedridden at home or hospitalized).

### Statistical analyses.

Proportions are presented as percentages, and continuous data as medians with interquartile ranges (IQR). Participants were followed from a positive PCR test to the last ELISA. Survival analysis was applied to handle the differences in time from PCR-testing to the last result of ELISA antibody-detection. Time since PCR was defined in days. Where multiple serum samples were conducted, the time until the last serum sample was used. An event was defined as a serum sample with no detection of SARS-CoV-2 antibodies. Where the last serum samples were positive for SARS-CoV-2 antibodies, the participant was censored afterwards. Survival times and event probability plots were used to investigate the probability of not having detectable antibodies over time, thus the risk of being seronegative a defined time after a positive PCR test. Risk factors (age, sex, body mass index [BMI], disease severity, smoking, having contact with patients) for not having detectable antibodies after a positive PCR were investigated in uni- and multivariable Cox proportional hazards models yielding hazard ratio (HR) and 95% confidence intervals (CI). The proportional hazard assumptions were tested by plotting Schoenfeld residuals against time. All analyses were conducted in the statistical software R version 3.6.1.

### Data availability.

The data presented in this study are available on request to the corresponding author. The data are not publicly available, due to Danish legislation.
